# Poor Sleep Quality Is the Risk Factor for Central Serous Chorioretinopathy

**DOI:** 10.1155/2018/9450297

**Published:** 2018-08-01

**Authors:** Yuying Ji, Miaoling Li, Xiongze Zhang, Yuting Peng, Feng Wen

**Affiliations:** State Key Laboratory of Ophthalmology, Zhongshan Ophthalmic Center, Sun Yat-sen University, 54 South Xianlie Road, Guangzhou 510060, China

## Abstract

**Purpose:**

Whether sleep disturbance is related with central serous chorioretinopathy (CSC) is still in controversy. This study is designed to investigate sleep status in CSC using definite and well-established methods.

**Methods:**

A total of 134 CSC patients and 134 age- and sex-matched normal controls were recruited in the study. Demographic data were collected through a questionnaire. Body mass index (BMI) was calculated by weight divided by height squared. The Pittsburgh Sleep Quality Index (PSQI) and Epworth Sleepiness Scale (ESS) were administered to all subjects to assess the sleep quality and daytime sleepiness, respectively. Depression Anxiety Stress Scales 21-item version (DASS-21) was also used to evaluate the emotion status as a positive control. Poor sleep quality was defined as PSQI > 5 and sleep apnea tendency as ESS > 10. Positive criteria scores were ≥10 for depression, ≥8 for anxiety, and ≥15 for stress.

**Results:**

There was no significant difference of BMI between the two groups (*p*=0.075). The prevalence of poor sleep quality (58.2% versus 23.9%; *p* < 0.001) in CSC patients was significantly higher than normal. Specifically, CSC patients presented worse performance in certain components of sleep quality, that is, sleep latency, sleep duration, and sleep efficiency. More participants had stress (23.9% versus 3%, *p* < 0.001), depression (25.4% versus 10.4%; *p*=0.001), and anxiety (28.4% versus 14.9%; *p*=0.008) emotions in CSC than that in normal. No significant difference was observed in sleep apnea tendency. Through logistic regression analysis, CSC patients were more likely to be in poor sleep quality (*p* < 0.001; OR 3.608 (2.071–6.285)) and stress emotion (*p*=0.002, OR 6.734 (1.997–22.711)).

**Conclusion:**

Poor sleep quality is risk factor for CSC patients. Attention of sleep quality should be paid when treating them.

## 1. Introduction

Central serous chorioretinopathy (CSC) is a common disorder characterized by serous retinal detachment (SRD) and/or retinal pigment epithelial detachment and often associated with subretinal fluid leakage [1, 2]. It ranks the fourth among the nonsurgical posterior segment diseases [3]. Several independent studies have reported that it occurs most frequently in mid-age with the peak age around 45 years old and a male preponderance [4, 5]. The exact mechanism of CSC is still unknown. Certain events have been reported to be risk factors for CSC, such as stress [6], type A behavior [7], hypertension [8], sleep disturbance [9, 10], or sleep apnea [11, 12]. However, whether sleep disturbance is associated with CSC is in controversy, and sometimes it is confused with sleep apnea. In order to have a better understanding and guidance for the cause and treatment of the disease, we conducted this survey to collect sleep situation of CSC patients and analyze their association with the disease.

## 2. Materials and Methods

### 2.1. Compliance

The study procedure was approved by the Ethics Committee of the Zhongshan Ophthalmic Center, Sun Yat-sen University. The protocol was conducted according to the Declaration of Helsinki. After explaining the purpose and procedure of the study, all the subjects signed the written informed consent.

### 2.2. Inclusion and Exclusion Criteria

Treatment-naive CSC patients were enrolled from the outpatient in the Zhongshan Ophthalmic Center. They all underwent physical examination including height, weight, and blood pressure. Ophthalmic examinations were taken in all the CSC patients, including visual acuity, slit-lamp biomicroscopy, direct ophthalmoscopy, color fundus image, and fundus fluorescence angiography (FFA, FFA 450 plus, Zeiss, Germany).

An inclusion criterion for acute CSC was focal serous retinal detachment involving macula with one or more leakage in FFA within 6 months. For chronic patients, inclusion criteria were persistent subretinal fluid involving macula for at least 6 months or serous retinal detachment with diffuse atrophy and decompensation of retinal pigment epithelium (RPE) (also referred to as diffuse retinal pigment epitheliopathy) with/without gradual, indistinct RPE leakage on FFA [2, 13, 14].

Volunteers enrolled from oral and poster advertisements around our hospital were included as the control group. They were age and sex matched at a ratio of 1 : 1. They all underwent ophthalmic examinations including visual acuity, slit-lamp biomicroscopy, and direct ophthalmoscopy to rule out the ophthalmic diseases. Physical examination including height, weight, and blood pressure was taken in all the participants.

Participants with other fundus diseases such as rhegmatogenous retinal detachment, choroidal neovascularization, polypoidal choroidal vasculopathy (PCV), retinal vein occlusion, diabetic retinopathy, Vogt–Koyanagi–Harada, optic disc pit, and scleritis were excluded. Participants with a history of trauma or surgery within one month were also excluded. Participants with hypertension (when systolic pressure was consistently greater than 140 mmHg or when diastolic pressure was consistently 90 mmHg or more) and alcohol, tobacco, or steroid using history were also excluded.

Finally, a total of 134 CSC patients and 134 age- and sex-matched controls were included in our study.

### 2.3. Criteria for Evaluations

Body mass index (BMI) was calculated using the formula in which weight in kilograms is divided by height in meters squared. Participants were divided into four groups (underweight, healthy, overweight, and obese) according to the BMI score. Underweight was defined as BMI score <18.5, healthy was 18.5 to 24.99, overweight was 25.0 to 29.99, and obese was 30 or higher.

#### 2.3.1. PSQI

The Pittsburgh Sleep Quality Index (PSQI) is a self-report questionnaire that assesses sleep quality over a 1-month time interval [15], developed by researchers in the University of Pittsburgh (http://www.psychiatry.pitt.edu/node/8240). The PSQI was intended to be a standardized sleep questionnaire for clinicians and researchers. The PSQI has well-established reliability and validity and is widely used in clinical research. The measure consists of 19 individual items designed to assess seven components of sleep: subjective sleep quality, sleep latency, sleep duration, habitual sleep efficiency, sleep disturbances, use of sleeping medication, and daytime dysfunction. These items generate a global score with a range of 0–21. More global sleep score indicated poorer sleep quality. A global sleep score above 5 (not including 5) was defined as poor sleep quality in our study.

#### 2.3.2. ESS

The Epworth Sleepiness Scale (ESS) is a scale introduced in 1991 in Australia. The scale was designed to measure daytime sleepiness and was helpful in diagnosing sleep disorders [16]. The questionnaire described eight different situations in daily life and asked the subject to rate his or her probability of falling asleep on a scale of increasing possibility from 0 to 3. The cumulative score in eight questions was the total score. A number below 10 was considered to be normal and above 10 indicated a tendency for sleep apnea.

#### 2.3.3. Emotion Status Evaluation

Stress was thought to be closely related with CSC. So, we used Depression Anxiety Stress Scales 21-item version (DASS-21) to evaluate the emotion status as a positive control. DASS, developed by Lovibond and Lovibond [17], was made up of 42 self-report items designed to measure the three related negative emotional states of depression, anxiety, and tension/stress. The DASS-21 was a short version of DASS that contains a 21-question assessment and had the same internal consistency and concurrent validity as DASS. It included seven questions. The scores were calculated by adding up the scores for each item in the section. Each of these was rated on a four-point Likert Scale of frequency or severity of the participants' experiences over the last week with the intention of emphasizing states over traits. These scores ranged from 0, meaning that the client believed the item “did not apply to them at all,” to 3 meaning that the client considered the item to “apply to them very much or most of the time.” The definition of positive in emotion was 10 and above for depression, 8 and above for anxiety, 15 and above for stress.

### 2.4. Survey Quality Control

All the investigators were medical professionals. They were trained about the survey components and the survey process. Participants filled in the questionnaire by themselves. Investigators supervised the circumstances and the time. After the survey was done, investigators made sure that participants had already answered all the questions. If there were any obvious mistakes and omit options, investigators pointed out and explained to participants to help complete the survey.

### 2.5. Statistical Analysis

The SPSS 22.0 software (SPSS Inc., Chicago, IL, USA) was used to perform the statistical analysis. The continuous variables were expressed in the form mean ± standard deviation in normal distributed sample and in frequency and percentage form in nonnormally distributed sample. The independent *t*-test and Mann–Whitney *U* test were used for the comparison of continuous variables. The chi-square test and Kruskal–Wallis *H* test were used to compare the categorical variables. Pearson correlation analysis was used to evaluate the relationship between PSQI and DASS-21 scores. Logistic regression analysis was used to compare the risk factors between CSC and normal controls. *p* < 0.05 was considered to be statistically significant.

According to the positive rates in CSC patients and normal controls, the set of alpha was 0.01, permissible error was less than 0.01, the sample size should be larger than 133 to estimate the differences.

## 3. Results

The demographic data of 134 CSC patients and 134 normal controls are shown in Table 1. Participants were predominantly male with a mean age of 44.57 ± 6.29 years for CSC patients and 44.36 ± 5.83 for normal controls (*p*=0.777). The mean BMI is 22.51 ± 2.38 for CSC patients and 22.32 ± 2.80 for controls with an insignificant difference (*p*=0.075).

Among all the participants, 20 CSC patients and 16 normal controls had gone to bed after 24:00, with the last one at 2 a.m. in the morning. Ten CSC patients and 10 normal controls had got up early in the morning; the earliest is around 4 a.m. in the morning. As shown in Table 1, no differences were observed in the distribution of sleep and wake up time between CSC patients and normal controls (*p*=0.161 for sleep time; *p*=0.333 for wake up time).

The PSQI, ESS, and DASS scale results are shown in Table 2. According to the cutoff criterion, 58.2% of CSC patients and 23.9% of normal controls had poor sleep quality. There were 23.9% of CSC patients and only 3% of normal controls who were in stress emotion. The percentage of poor sleep quality and stress emotion in CSC patients was significantly higher than that in normal controls (*p* < 0.001). There were also more participants who had depression and anxiety emotion in CSC than in normal controls with a statistically different *p* value (*p*=0.001 for depression, *p*=0.008 for anxiety). No significant difference was observed in sleep apnea tendency between two groups. Through Pearson correlation analysis, a positive correlation existed between PSQI and DASS-21 scores in both groups, as shown in Table 3.

The details in four out of seven subscale dimensions of PSQI are shown in Table 4. Compared with controls, CSC patients reported more percentage in bad subjective sleep quality (*p* < 0.001), longer time of sleep latency (*p* < 0.001), shorter sleep duration (*p* < 0.001), and less sleep efficiency (*p* < 0.001), indicating they were worse sleepers than normal controls.

Table 5 shows the results of logistic regression analysis. It showed that CSC patients were more likely to be in poor sleep quality (*p* < 0.001; OR 3.608 (2.071–6.285)) and stress emotion (*p*=0.002; OR 6.734 (1.997–22.711)). But depression did not increase the risk (*p*=0.378; OR 1.563 (0.579–4.218)) neither did the anxiety (*p*=0.390; OR 0.669 (0.267–1.673)).

## 4. Discussion

Our case-control study involving a relatively large sample size reveals that CSC patients performed worse in sleep function and emotion regulation in China, suggesting that poor sleep quality and stress are risk factors for CSC. But sleep apnea is not associated with CSC.

Our findings expand the observations of sleep status in CSC patients. Setrouk et al. [18] conducted a retrospective case-control study, and there was no significant difference in PSQI scores between chronic CSC and other patients with a limited sample size of 29. A retrospective study carried out in Korea in 2012 including 113 CSC patients indicated that sleep disturbance defined as “subjects were questioned whether they awoke feeling tired or if they experienced fatigue during the day” was strongly associated with CSC [9]. This definition was indistinct. We use a standard assessment of sleep quality by the PSQI questionnaire, which combined the quality and quantity of sleep, graded sleep according to the scores, and was a validated, reliable tool. Through our study, we find that CSC patients in China had worse sleep quality, especially in dimension of subjective sleep quality, sleep latency, sleep duration, and sleep efficiency. These findings provide more information about CSC patients. In a meta-analysis conducted by Liu et al. [10], three studies used for the “sleep disturbance” were not under the same criteria. Two of the three studies were actually related with sleep apnea, and one study conducted by Brodie et al. [11], as we previous described, had ruled out the obstructive sleep apnea as a risk factor after being adjusted for BMI. The remaining study used a vague definition for “sleep disturbance” as discussed before.

Our study does not evaluate shift work or irregular hours which might have an impact on CSC through circadian disruption [18–20]. But from the sleep and wake up time of our participants, no obvious circadian disruption is observed.

Our study does not suggest that patients with CSC are more likely to have sleep apnea. In a meta-analysis conducted by Wu et al. [21], it was suggested that patients with CSCR is more likely to have sleep apnea. But the diagnostic criteria were not the same in the included six studies. And BMI, a known risk factor for sleep apnea [22, 23], was not investigated in five out of the six studies. When Brodie et al. [11] corrected the BMI, no significant relationship was found between obstructive sleep apnea and CSC. Although sleep apnea is not diagnosed through ESS, scores over 10 are indicating a tendency for sleep apnea. Our result is similar with Brodie's result. This might be attributed to the BMI, an important risk factor for obstructive sleep apnea. Most patients in our study have a normal BMI as the normal controls.

Our study further extends the understanding of emotion status in CSC patients. Data from DASS-21 show that prevalence of depression, anxiety, and stress emotion was significantly higher in CSC patients compared to that in normal controls. Psychological stress and type A behavior were found to be associated with onset of CSC [6, 7]. Furthermore, in recent years, antianxiety drugs [24], psychopharmacologic medication use [25], and stress [19] were found to be risk factors for CSC, and a history of psychiatric illness increased the CSC recurrence risk [26]. The scores and grading in our DASS-21 test provide further evidence from CSC patients' subjective feelings, suggesting that many CSC patients have unhealthy emotions especially the stress.

The cause and effect relationship between sleep, emotion, and CSC is not clear. The following are our speculations. First, glucocorticoid is involved in the development of central serous chorioretinopathy [27]. Increased levels of 24-hour urine corticosteroid and serum cortisol are found in CSC patients [28, 29]. Under certain physiological stress, the adrenal gland secretes glucocorticoid in response to the signal from the hypothalamus and may contribute to the pathogenesis of the disorder [30]. Second, depression will influence the effects of peptides on sleep EEG and hormone secretion [31], and this could explain that there are more patients with the depression emotion than controls. These studies suggest that attention should be paid to patients' emotions and sleep quality when treating CSC patients.

There are some limitations of this study. First, we do not include all the possible risk factors for CSC such as *Helicobacter pylori* and shift work to be more comprehensive. Second, the patients enrolled in this study come from one ophthalmic center, and multicenter analysis will be more persuasive and thorough.

## 5. Conclusion

The study reveals that poor sleep quality and stress emotion are risk factors for CSC patients in China. It may indicate that attention should be paid to patients' sleep and emotions to achieve better treatment outcome.

## Figures and Tables

**Table 1:** Comparison between CSC patients and normal controls.

	CSC (*n*=134)	Control (*n*=134)	*p*
Age (mean ± standard deviation)	44.57 ± 6.29	44.36 ± 5.83	0.777 (independent *t*-test)
Sex			
Male	110 (82.1%)	110 (82.1%)	1 (chi-square test)
Female	24 (17.9)	24 (17.9)	
BMI	22.51 ± 2.38	22.32 ± 2.80	0.075 (Kruskal–Wallis *H* test)
Underweight	6 (4.5%)	8 (5.97%)	
Healthy	106 (79.1%)	114 (85.07%)	
Overweight	22 (16.4%)	12 (8.96%)	
Sleep time			
20:00–21:00	4 (2.99%)	10 (7.46%)	0.161 (Kruskal–Wallis *H* test)
21:01–24:00	110 (82.10%)	108 (80.60%)	
0:01–2:00	20 (14.93%)	16 (11.94%)	
Wake up time			
4:00–5:00	10 (7.46%)	10 (7.46%)	0.333 (Kruskal–Wallis *H* test)
5:01–9:00	118 (88.06%)	112 (83.58%)	
9:01–10:00	6 (4.48%)	12 (8.96%)	

CSC, central serous chorioretinopathy; BMI, body mass index.

**Table 2:** Sleep and emotion status in CSC patients and controls.

	Results	Score	CSC	Control	*p* (chi-square test)
PSQI	−	≤5	56 (41.8%)	102 (76.1%)	**<0.001**
	+	>5	78 (58.2%)	32 (23.9%)	

ESS	−	≤10	120 (89.6%)	122 (91.0%)	0.680
	+	>10	14 (10.4%)	12 (9.0%)	

Depression	−	<10	100 (74.6%)	120 (89.6%)	0.001
	+	≥10	34 (25.4%)	14 (10.4%)	

Anxiety	−	<8	96 (71.6%)	114 (85.1%)	0.008
	+	≥8	38 (28.4%)	20 (14.9%)	

Stress	−	<15	102 (76.1%)	130 (97.0%)	**<0.001**
	+	≥15	32 (23.9%)	4 (3.0%)	

CSC, central serous chorioretinopathy; PSQI, Pittsburgh Sleep Quality Index; ESS, Epworth Sleepiness Scale.

**Table 3:** Correlation between PSQI and DASS-21 scores in CSC and control groups (Pearson correlation analysis).

	PSQI (CSC)		PSQI (control)	
	*r*	*p*	*r*	*p*
Depression	0.350	<0.001	0.529	<0.001
Anxiety	0.235	0.006	0.577	<0.001
Stress	0.369	<0.001	0.588	<0.001

CSC, central serous chorioretinopathy; PSQI, Pittsburgh Sleep Quality.

**Table 4:** Subscale of sleep features in CSC and controls.

	CSC		Controls		*p*
	*n*	%	*n*	%	
Subjective sleep quality					
0-very good	14	10.4	30	22.4	**<0.001** (Kruskal–Wallis *H* test)
1-fairly good	74	55.2	86	64.2	
2-fairly bad	24	17.9	18	13.4	
3-very bad	22	16.4	0	0	
Sleep latency >15 min	116	86.6	86	64.2	**<0.001** (chi-square test)
Sleep duration					
>7 h	46	32.8	94	70.1	**<0.001** (Kruskal–Wallis *H* test)
6-7 h	42	31.3	28	20.9	
5-6 h	28	20.9	6	4.5	
<5 h	18	13.4	6	4.5	
Sleep efficiency					
≥85%	66	49.3	110	82.1	**<0.001** (Kruskal–Wallis *H* test)
75%–84%	30	22.4	16	11.9	
65%–74%	20	14.9	2	1.5	
<65%	18	13.4	6	4.5	

CSC, central serous chorioretinopathy.

**Table 5:** The logistic regression of risk factors in CSC.

	Odds ratio	95% confidence interval	*p* value
PSQI ≥ 5	3.608	2.071–6.285	<0.001
Stress (+)	6.734	1.997–22.711	0.002
Depression (+)	1.563	0.579–4.218	0.378
Anxiety (+)	0.669	0.267–1.673	0.390

CSC, central serous chorioretinopathy; PSQI, Pittsburgh Sleep Quality Index.

## Data Availability

The questionnaire data used to support the findings of this study are restricted by the Ethics Committee of the Zhongshan Ophthalmic Center in order to protect patient privacy. Data are available from Feng Wen (wenfeng208@foxmail.com) for researchers who meet the criteria for access to confidential data.

## Abbreviations

BMI: Body mass index
CSC: Central serous chorioretinopathy
DASS: Depression Anxiety Stress Scales
ESS: Epworth Sleepiness Scale
FFA: Fundus fluorescence angiography
PCV: Polypoidal choroidal vasculopathy
PSQI: Pittsburgh Sleep Quality Index
RPE: Retinal pigment epithelium
SRD: Serous retinal detachment.
